# Impact of the 2018 Japan Floods on Methotrexate and Antirheumatic Drug Prescriptions: A Longitudinal Analysis of the Japanese National Database

**DOI:** 10.31662/jmaj.2025-0258

**Published:** 2025-11-21

**Authors:** Genki Kidoguchi, Shuhei Yoshida, Tomohiro Sugimoto, Shintaro Hirata, Masatoshi Matsumoto

**Affiliations:** 1 Department of Clinical Immunology and Rheumatology, Hiroshima University Hospital, Kasumi, Minami-ku, Hiroshima, Japan; 2 Department of General Medicine, Hiroshima University Hospital, Kasumi, Minami-ku, Hiroshima, Japan; 3 Department of Community-Based Medical System, Graduate School of Biomedical and Health Sciences, Hiroshima University Hospital, Kasumi, Minami-ku, Hiroshima, Japan

**Keywords:** disaster, mental stress, methotrexate, rheumatic diseases, 2018 Japan floods

## Abstract

**Introduction:**

Most rheumatic diseases are caused by a complex interplay of genetic, physical, and environmental factors. Large-scale disasters affect all of these factors; however, their impact on rheumatic diseases is unknown. We aimed to investigate changes in antirheumatic drug prescriptions among victims and non-victims after the 2018 Japan Flood: the second largest water-related disaster in Japan.

**Methods:**

In this retrospective cohort study, we used data from the Japanese National Database of Health Insurance Claims, which included information on all drugs prescribed by physicians. We included all cases of prescription at medical institutions in disaster-stricken areas between July 2017 and June 2019. The newly initiated prescription of methotrexate (MTX, 2-mg tablets or capsules), which has been exclusively approved for rheumatoid arthritis, juvenile idiopathic arthritis, or psoriatic arthritis/psoriasis in Japan, and other antirheumatic drugs within the first year after the disaster were evaluated for government-certified disaster victims and non-victims. Baseline characteristics and MTX prescription status in the pre-disaster period were also assessed to compare the groups.

**Results:**

In the pre-disaster period, no significant association was found between victim status and MTX prescription. The number of individuals who had not been prescribed MTX before the disaster was 4,973,401, including 31,006 victims. Among them, 14,908 (including 110 victims) had a history of MTX prescription after the disaster. In the MTX-naive group, new MTX prescriptions within one year after the disaster were significantly more frequent in victims than in non-victims (age- and sex-adjusted hazard ratio: 1.83; 95% confidence interval: 1.37-2.46). Similarly, a non-significant increase in prescriptions for conventional synthetic/biological disease-modifying antirheumatic drugs was observed.

**Conclusions:**

Victims of the 2018 Japan Flood were more likely to be prescribed MTX for the first time.

## Introduction

Most rheumatic diseases result from a complex interplay of genetic, physical, psychological, and environmental factors. For instance, rheumatoid arthritis (RA) is associated with viral and bacterial infections, changes in gut microbes, poor oral hygiene, obesity, and psychological stress ^(1), (2)^.

Currently, global climate change is the cause of unprecedented large-scale natural disasters. These affect various aspects of human health through environmental, physical, and psychological factors, as well as health care delivery systems. Previous studies have demonstrated increased initiation of specific medications among victims with cognitive decline, sleep disorders, irritable bowel syndrome, and migraine ^(3), (4), (5), (6)^. Based on these findings, examining new prescriptions of antirheumatic drugs before and after a natural disaster may reflect the impact of disasters on changing health care needs for rheumatic diseases after a disaster. However, no large-scale studies have examined disaster-related changes in new antirheumatic drug prescriptions. In recent years, Japan has experienced a series of large-scale disasters, including the Great East Japan Earthquake. Therefore, clinicians must recognize how disasters affect rheumatic diseases through changes in medication prescriptions.

Studies examining disaster-related changes in medication prescriptions have been limited to patients with RA. A previous study investigated the association between the Great East Japan Earthquake and RA activity in a cohort of 53 patients and revealed exacerbation of disease activity after the disaster ^(7)^. Another study observed the influence of natural disasters such as earthquakes, typhoons, and heavy rains on 192 patients with RA and reported the deterioration of physical function 1 and 6 months after the disaster ^(8)^. However, these were small-scale and case-based studies performed without a control group and thus could not establish causal relationships between disasters and changes in rheumatic diseases.

Therefore, the present study examined changes in antirheumatic drug prescriptions using population-based large-scale cohort data. We hypothesized that disaster victims would be more likely to receive new prescriptions for antirheumatic drugs. The data used in this study corresponded to the census data of national health insurance claims and were analyzed one year before and after the 2018 Japan Floods. In the analysis, we compared new prescriptions of antirheumatic drugs between government-certified victims and non-victims.

## Materials and Methods

### Study design and data collection

After obtaining permission to use the data from the Ministry of Health, Labor, and Welfare (Permission number 12232), this retrospective cohort study used data from the Japanese National Database of Health Insurance Claims and Specific Health Checkups (NDB). The NDB is one of the several government-maintained nationwide health care-related databases in Japan and includes information on all prescription drugs dispensed in the country because all Japanese residents are covered by public health insurance. Japan has a universal health insurance system, and the NDB contains all the health service claims made by every individual who visited any medical institution.

### Setting

Between June 28 and July 8, 2018, western Japan experienced torrential rainfall that triggered landslides and led to river overflowing. In terms of their destructive magnitude, the 2018 Japan Floods were the second-largest water-related disaster in the recorded history of Japan, surpassed only by the 2011 Great East Japan Earthquake. The aftermath included 263 fatalities, 484 injuries, and eight missing individuals. Furthermore, 6,783 homes were destroyed and another 44,327 were damaged. Hiroshima, Okayama, and Ehime were the prefectures that were hit the hardest, accounting for approximately 90% of the total deaths. The estimated cost of the damage reached approximately $12.66 billion US dollars (converted at a central rate of $1 US dollar to ¥111.37 Japanese yen, based on the average rate in the Tokyo Market for July 2018).

Hiroshima, Okayama, and Ehime prefectures were selected as target areas for the location of medical institutions. The survey period was set between July 2017 and June 2019, which corresponds to a period of one year before and one year after the disaster.

### Participants

The participants in this study were individuals aged ≥20 years who visited medical institutions located in the three target prefectures and had health insurance claims issued during the specified survey period. Among the participants, those identified as disaster victims by the local government were labeled as such in this study. The remaining participants were categorized as non-victims and analyzed separately.

The data set used for this study, originally compiled to assess outcomes such as long-term care costs, did not include detailed diagnostic codes, such as those from the International Classification of Diseases, 10th Revision (ICD-10), limiting definitive disease identification for this secondary analysis. Therefore, we adopted a strategy of defining our study populations based on prescription patterns of disease-specific medications. This approach allows an analysis of a comprehensive, real-world cohort of all health care users captured by the NDB rather than a pre-selected clinical population. We focused on methotrexate (MTX; 2 mg) prescriptions because this formulation is specifically indicated for rheumatic diseases in Japan, and, as detailed in a later subsection (“Targeted drugs”), the majority of these prescriptions are considered to be for the treatment of RA.

Two distinct groups were included in this study. The first group consisted of participants who had no history of MTX prescriptions before the disaster (the MTX-naive group), enabling us to identify those who had been newly prescribed MTX after the disaster. The second group, for the analysis of secondary outcomes, consisted of participants with a history of MTX prescription after the disaster, from whom participants with a history of other antirheumatic drugs (detailed in the “Targeted drugs” subsection) prescriptions before the disaster were excluded (the MTX-user group). This enabled us to identify those who were prescribed antirheumatic drugs in addition to MTX after the disaster. We excluded patients aged <20 years from the target population.

### Definition of disaster victims

During the 2018 Japan Flood, the Japanese government announced that certified victims would be fully exempt from medical insurance co-payments (10%-30% of the total medical expenses) ^(9)^. Therefore, we defined a “disaster victim” as a person listed as a government-certified disaster victim in health insurance claims issued after the disaster. However, people whose medical expenses had already been exempted by the government, such as those under public livelihood protection and atomic bomb survivors, were not eligible for disaster-related exemption; therefore, they were categorized as non-victims even if they were affected.

Disaster victims were certified by the local government of their residential municipality. The criteria for a certified disaster victim fell into one of the following categories: (1) the residential house was completely or partially destroyed, burned down, flooded above the floor level, or similarly damaged; or (2) family member who had financially supported the person was killed or suffered severely or was missing. The validity of this definition of disaster victims is supported by its use in multiple studies investigating the impact of this disaster on different diseases using the same data set ^(3), (4), (5), (6)^.

### Targeted drugs

Many antirheumatic drugs are covered by insurance and have indications for treating other diseases. In Japan, oral MTX is available in two formulations (2 mg and 2.5 mg), each indicated for different diseases. The 2.5-mg formulation is specifically indicated for trophoblastic diseases and chronic lymphocytic leukemia, whereas the 2-mg formulation is exclusively approved for RA, juvenile idiopathic arthritis (JIA), and diseases related to psoriasis (Pso), such as PsA and pustular Pso. Although some patients with JIA transition to adult care and are treated with MTX, this patient population is considerably smaller than that with RA. Insurance coverage for MTX prescriptions for Pso in Japan commenced in December 2018. In addition, even if MTX was used after the insurance coverage began, the proportion of prescriptions for Pso is expected to be much smaller than that for RA, according to the disease prevalence: RA, 0.75%; Pso, 0.34% ^(10), (11)^. Furthermore, MTX is prescribed as the initial treatment for 85% of patients with RA, whereas only 39.1% of patients with Pso receive it during their treatment course ^(12), (13)^. These epidemiological data suggest that the majority of MTX prescriptions are for the treatment of RA.

In addition, we extracted data on antirheumatic drugs used for the treatment of rheumatic diseases, such as RA, in Japan as of 2019: conventional synthetic disease-modifying antirheumatic drugs (csDMARDs; including salazosulfapyridine, iguratimod, tacrolimus, bucillamine, and mizoribine), biological DMARDs (bDMARDs; including infliximab, etanercept, adalimumab, certolizumab pegol, golimumab, abatacept, and tocilizumab), and glucocorticoids (oral and injectable formulations). We did not include other immunosuppressants, such as mycophenolate mofetil, azathioprine, or cyclophosphamide. Although these drugs are used for some rheumatic diseases, they also have broad indications for non-rheumatic conditions, including organ transplantation, in Japan. In a data set lacking specific diagnostic codes, including these less-specific agents could introduce significant measurement error. For the secondary analysis of the MTX-user cohort, however, our definition of csDMARDs included agents such as tacrolimus and mizoribine, which are considered clinically relevant second-line options for diseases for which MTX is approved under Japanese health insurance, such as RA.

### Outcomes

We examined new prescriptions of targeted drugs after the disaster to evaluate changes in antirheumatic drug use. We focused on new prescriptions rather than existing ones because the continuation or discontinuation of existing prescriptions could be influenced by various factors such as health care access during disasters, whereas new prescriptions reflect medical needs more directly. The month in which the new prescription of the targeted drug was issued to the participant was identified in the database. The primary outcome of this study was the occurrence of new MTX prescriptions during the one-year period after the disaster in the MTX-naive group. The secondary outcome was defined as the new occurrence of prescriptions for csDMARDs, bDMARDs, and glucocorticoids at a dosage of 1 mg or more during the one-year period after the disaster among participants in the MTX-user group.

### Statistical analyses

We performed a chi-square test to compare the binary and categorical variables between the victim and non-victim groups. In addition, the absolute standardized difference (ASD) was calculated to assess baseline imbalances, with an ASD > 0.1 considered meaningful. In accordance with the NDB Japan rule, data masking is applied when the number of observations is 10 or less. For the chi-square test in Table 1, we proceeded with the test because the expected value was above 10. In addition, we analyzed the differences in the incidence rates of MTX, DMARDs, and glucocorticoids between the two groups using the Kaplan-Meier method with the log-rank test. We used the Cox proportional hazards model adjusted for sex and age to quantitatively measure the impact of the disaster on the probability of new MTX and other drug prescriptions after ensuring, with the Schoenfeld residuals method, that the proportional hazards assumption was met. The follow-up period was defined as up to one year after disaster onset or until the first prescription of MTX for primary outcomes and up to one year after disaster onset or until the first prescription of other antirheumatic medications for secondary outcomes. Furthermore, analyses were conducted to verify whether there was an inherent likelihood of disaster victims receiving MTX prescriptions more frequently than non-victims did before the disaster. We conducted logistic regression analyses for each period before and after the disaster after adjusting for age and sex to examine the association between disaster status and the incidence of total MTX prescriptions. In addition, we tested for interactions between the time variable (pre-disaster or post-disaster) and disaster-affected status to assess whether the effect of disaster victim status on MTX prescriptions differed significantly between the pre-disaster and post-disaster periods. All statistical analyses were performed using STATA/MP software (version 17; Stata Corp, 2019). Two-sided p < 0.05 were considered statistically significant.

**Table 1.** Baseline Characteristics of MTX-Naive and MTX-User Patients.

		MTX-naive			MTX-user		
		Victims	Non-victims	ASD	Victims	Non-victims	ASD
All participants, n		31,006	4,942,395		110	14,798	
Age classification, n (%)	20-44	6,668 (21.5%)	1,710,176 (34.6%)	0.295	^a^ (^a^)	1,067 (7.2%)	NC
	45-64	8,103 (26.1%)	1,451,712 (29.4%)	0.074	^b^ (^b^)	4,949 (33.4%)	NC
	65+	16,235 (52.4%)	1,780,507 (36.0%)	0.335	74 (67.3%)	8,782 (59.3%)	NC
Sex, n (%)	Male	13,528 (43.6%)	2,298,159 (46.5%)	0.058	31 (28.2%)	3,948 (26.7%)	0.034
	Female	17,478 (56.4%)	2,644,236 (53.5%)	0.058	79 (71.8%)	10,850 (73.3%)	0.034

Data were presented as n (%). ASD could not be calculated because the data were masked due to national regulations. An ASD of < 0.1 was considered to indicate a negligible difference between the groups. MTX-naive patients had no history of MTX prescription before the disaster, whereas MTX-user patients were defined as participants with a history of MTX prescription after the disaster, from whom those with a history of other antirheumatic drug prescriptions before the disaster had been excluded. ^a^Values less than 10 that were masked owing to the NDB Japan rules. ^b^Values over 10 that were masked due to NDB Japan rules. ASD: absolute standardized difference; MTX: methotrexate; NC: not calculated.

## Results

### Study population

**Figure 1.**
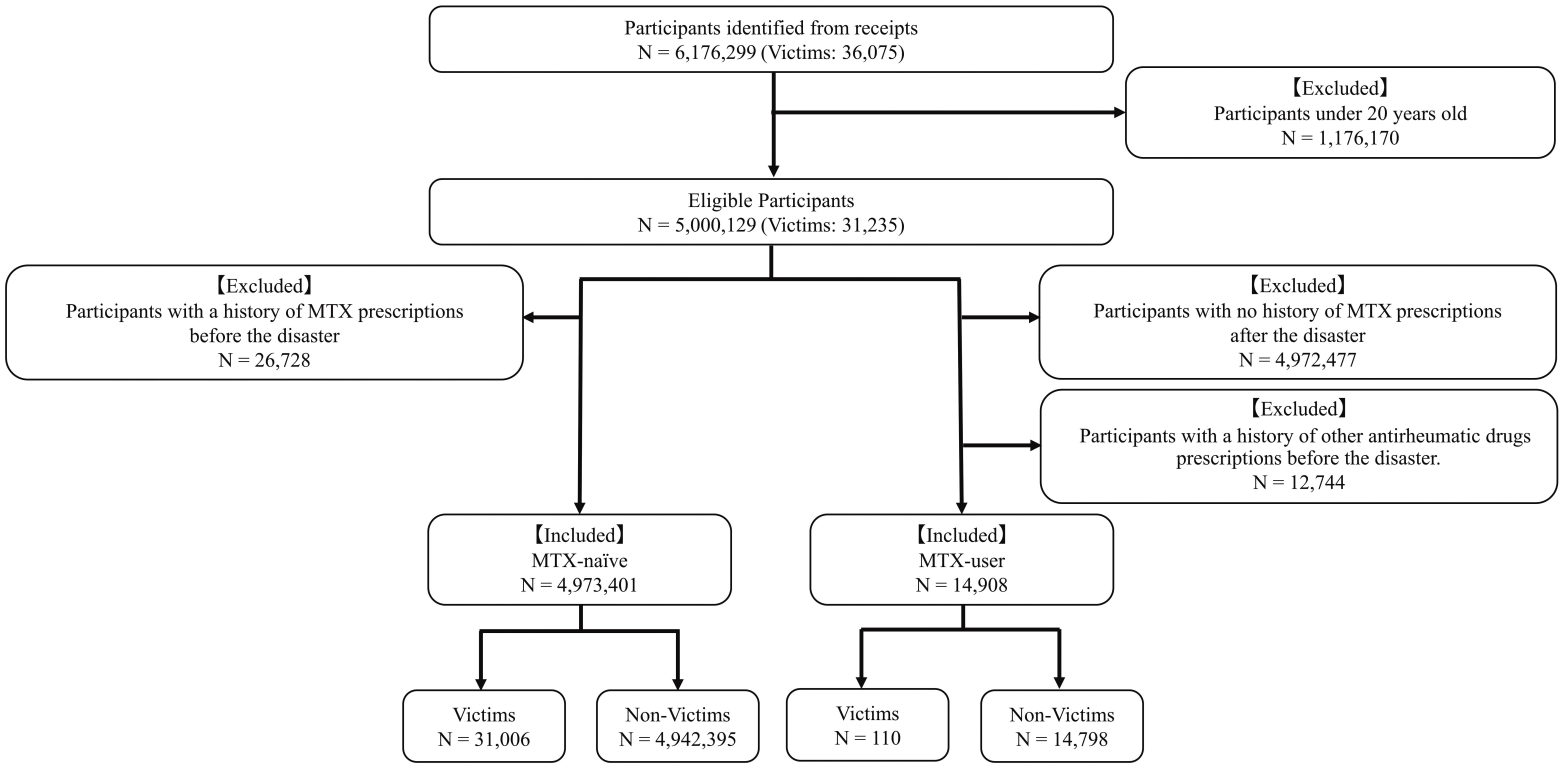
Flowchart of eligible and ineligible participants. The flowchart shows the selection of eligible and ineligible participants. MTX-naive patients were those who had no history of MTX prescriptions before the disaster, while MTX users were those who had a history of MTX prescriptions after the disaster. MTX: methotrexate.

A total of 1,556,403,905 prescription receipts were identified, leading to the identification of 6,176,299 participants. Of these, 1,176,170 participants under the age of 20 years were excluded. This resulted in 5,000,129 individuals being deemed eligible, of whom 4,973,401 were MTX-naive, having no history of MTX prescription before the disaster, whereas the MTX-user group comprised 14,908 individuals (Figure 1).

### Baseline characteristics

**Figure 2.**
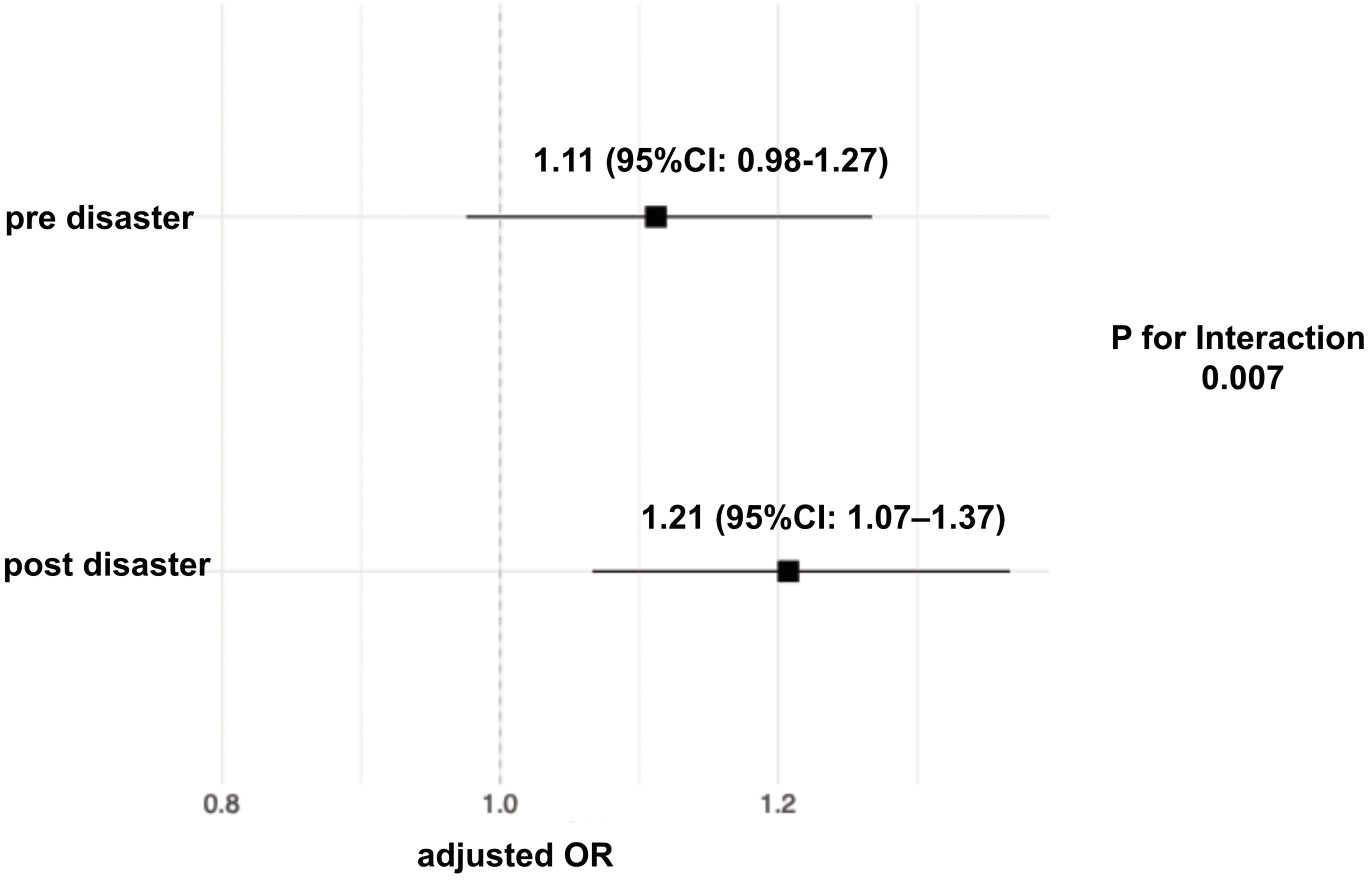
Time trend of adjusted OR of disaster victims for prescription of total MTX before and after the disaster. This analysis includes all eligible participants. This plot displays the adjusted OR for disaster victims regarding the association with total MTX prescriptions before and after the disaster. Pre-disaster users were included to evaluate trends prior to the disaster. The *p*-value for interaction indicates the association between the time variable (pre- or post-disaster) and disaster-affected status. MTX: methotrexate; OR: odds ratio.

Baseline characteristics of the eligible participants are detailed in Supplementary Table 1. An assessment using the ASD indicated meaningful imbalances in some baseline characteristics, as detailed in Table 1 and Supplementary Table 1. Overall MTX prescriptions were more frequent among disaster victims in the pre-disaster period. However, logistic regression analysis adjusted for age and sex revealed that the odds ratio for MTX prescriptions was 1.11 (95% confidence interval [CI]: 0.98-1.27) for the victims before the disaster and 1.21 (95% CI: 1.07-1.37) after the disaster (Figure 2), with no significant association between being a victim and receiving MTX prescriptions before the disaster.

The number of participants included in the MTX-naive group who had no history of MTX prescription was 4,973,401 of the 6,176,299 registered participants in the NDB. Table 1 indicates the baseline characteristics of the participants before the disaster. In total, 52.4% of victims and 36.0% of non-victims were aged >65 years old, and the percentages of women among victims and non-victims were 56.4% and 53.5%, respectively. The number of participants included in the MTX-user group who had a history of MTX prescriptions after the disaster was 14,908 of the 6,176,229 participants in the NDB. A total of 67.3% of victims and 59.3% of non-victims were aged over 65 years of age, and the percentages of women were 71.8% and 73.3%, respectively.

### New prescription of MTX

**Figure 3.**
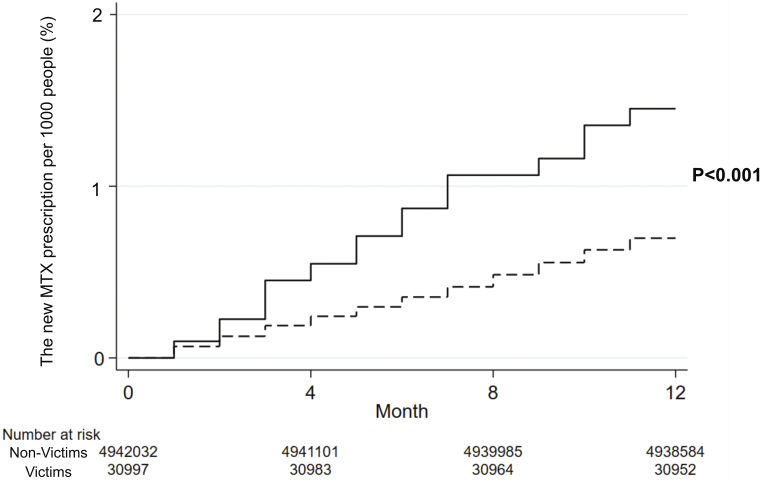
Kaplan-Meier failure curves for the participants newly prescribed with MTX. This analysis was performed on the MTX-naive group. The Kaplan-Meier curve illustrates the incidence of new MTX prescriptions within a span of 12 months after the disaster for victims and non-victims. MTX: methotrexate.

In the MTX-naive group, the incidence of new MTX prescriptions and Kaplan-Meier curves of the participants are shown in Table 2 and Figure 3, respectively. The results of log-rank tests showed that a new prescription of MTX within one year after the disaster occurred at a higher rate among disaster victims than among non-victims (p < 0.001).

**Table 2.** Incidence of New Prescription of MTX and Other Antirheumatic Drugs after the Disaster.

	Victims	Non-victims	p-Value
MTX, n (%)	54 (0.17%)	3,811 (0.08%)	p < 0.001
bDMARDs, n (%)	^a^ (^a^)	414 (2.8%)	p = 0.093
csDMARDs. n (%)	^a^ (^a^)	1,074 (7.3%)	p = 0.467
Glucocorticoid, n (%)	13 (11.8%)	1,547 (10,5%)	p = 0.641
Any of the antirheumatic drugs	23 (20.9%)	2,627 (17.8%)	p = 0.388

Data were presented as n (%). ^a^Values less than 10 that were masked owing to the NDB Japan rules. bDMARD: biological disease-modifying antirheumatic drug; csDMARD: conventional synthetic disease-modifying antirheumatic drug; MTX: methotrexate.

Supplementary Table 2 reveals the results of the risk evaluation of the new prescriptions for MTX using the Cox proportional hazards model. After being adjusted for age and sex, the hazard ratio (HR) of victims versus non-victims for new MTX prescription was 1.83 (95% CI: 1.37-2.46).

### Secondary outcome

**Figure 4.**
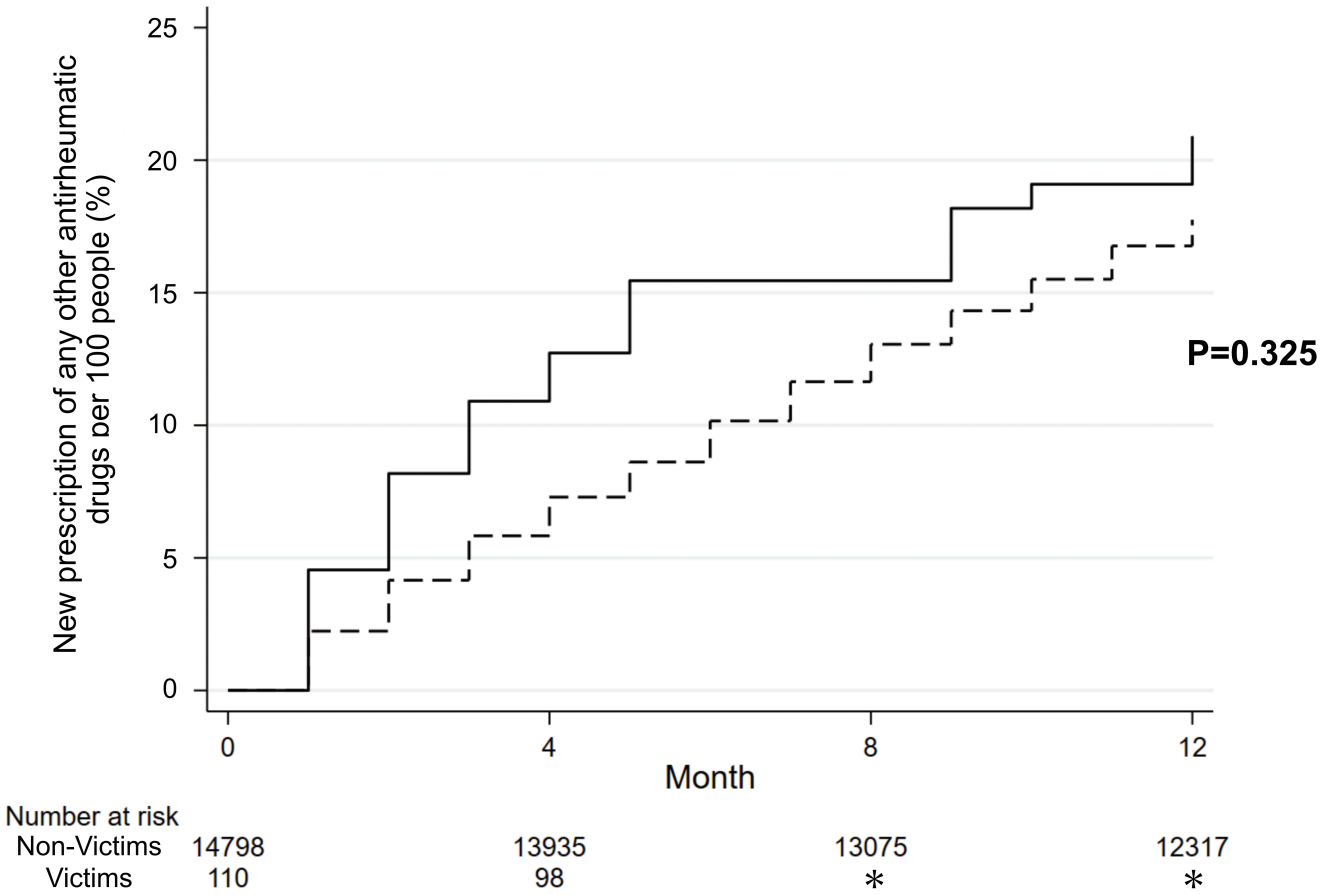
Kaplan-Meier failure curves for the participants newly prescribed with any other antirheumatic drugs. This analysis was performed on the MTX-user group. The Kaplan-Meier curve depicts the incidence of new other antirheumatic drug prescriptions (csDMARDs, bDMARDs, and glucocorticoids) among MTX-treated participants within a span of 12 months after the disaster among victims and non-victims. bDMARD: biological disease-modifying antirheumatic drugs; csDMARD: conventional synthetic disease-modifying antirheumatic drugs; MTX: methotrexate.

In the MTX-user group, Table 2 describes the prescription incidence and Figure 4 and Supplementary Figures 1-3 display the Kaplan-Meier curves for the MTX-user group according to the use of medications: bDMARDs in Supplementary Figure 1, csDMARDs in Supplementary Figure 2, glucocorticoids (oral and injectable) in Supplementary Figure 3, and any other antirheumatic drugs in Figure 4. Although the log-rank test showed no significant difference between the two groups (p = 0.325) for any of these three antirheumatic drugs, as detailed in Figure 4, a non-significant increase was observed.

Supplementary Table 3 displays the results of the risk evaluation of other antirheumatic drugs (csDMARDs, bDMARDs, and glucocorticoids) using the Cox proportional hazards model in the MTX-user group. The results revealed no significant differences between the victim and non-victim groups (p = 0.369). After adjusting for age and sex, the HR of victims versus non-victims for a new prescription of any of these three antirheumatic drugs was 1.21 (95% CI: 0.80-1.82, p = 0.369).

## Discussion

In this population-based cohort study, we revealed that disaster victims were more likely to be newly prescribed MTX after a disaster than non-victims. This finding was robust even after adjusting for age and sex. This study provides the first evidence from large-scale prescription data about changes in antirheumatic drug prescriptions after a disaster. However, prescriptions of bDMARDs, csDMARDs, and glucocorticoids among pre-disaster MTX users did not increase after the disaster.

Although MTX is widely accepted as the first-line treatment for RA in Japan and other countries, the 2-mg formulation is primarily prescribed for rheumatic diseases in Japan, with RA likely accounting for the majority of prescriptions. Therefore, the increased rate of new MTX prescriptions among victims suggests an association between becoming a disaster victim and an increased need for MTX-based therapy. This new need for MTX may reflect several clinical scenarios, including the onset of symptoms leading to a new diagnosis of a rheumatic disease, the exacerbation of a previously mild or unmedicated condition, or a necessary switch from another therapy, any of which could be triggered by the significant physical and mental burden and drastic environmental changes brought by the disaster. To the best of our knowledge, this is the first study to evaluate changes in antirheumatic drug prescriptions among disaster victims and compare new prescriptions of these drugs between certified victims and non-victims living in the affected areas. Considering the study design and the large-scale, population-based data, we believe that this study suggests a significant association between being a victim of a disaster and changes in antirheumatic drug prescriptions. These findings have significant implications for clinicians and policymakers, considering the increasing frequency of large-scale natural disasters worldwide. The recognition that being a victim of a disaster is associated with an increased need for antirheumatic drugs serves as a reminder to clinicians of the potential increase in such prescriptions after such events. Given the anticipated surge in demand for MTX and other antirheumatic drugs during and after disasters, the results will be useful for strategizing drug stockpiling in disaster-susceptible areas. Furthermore, another study that investigated the correlation between migraine episodes and natural disasters emphasized the criticality of pre-disaster preparedness ^(14)^.

In this study, no significant difference was found in the incidence of new prescriptions for bDMARDs, csDMARDs, or glucocorticoids between victims and non-victims among MTX users, although a trend toward a higher prescription rate was observed in the victim group. One potential explanation is that the disaster-related disruption of daily lives may have caused reluctance among patients to start new medications because of increased hospital visits, psychological stress, and potential side effects. Furthermore, the limited study period and number of MTX-treated patients may have reduced the statistical power to detect significant differences.

Our results are consistent with the biological basis of rheumatic disease development and exacerbation. A previous study showed that patients with RA exhibited elevated levels of inflammatory cytokines, such as interleukin (IL)-1β, IL-6, interferon-γ, and tumor necrosis factor-α, in response to stress compared with healthy individuals ^(15)^. These inflammatory cytokines are involved in the development and exacerbation of rheumatic diseases ^(16)^. Thus, it is reasonable that patients with rheumatic diseases may develop symptoms because of an increase in inflammatory cytokines after a disaster.

This study had several limitations. First, although MTX 2-mg prescriptions were specific to rheumatic diseases in Japan, we could not identify which rheumatic disease was being treated. The data set used, as detailed in the Methods section, was originally designed for different research objectives, lacking the diagnostic codes necessary for this secondary analysis. However, the unique prescription format for MTX 2 mg in Japan allows the extraction of prescriptions specifically for rheumatic diseases without relying on ICD-10 codes, thus ensuring considerable diagnostic accuracy. In addition, our secondary analysis included tacrolimus and mizoribine, which, despite being clinically relevant, also have non-rheumatic indications. This could have led to some misclassification of the secondary outcomes, and the results should be interpreted with this in mind. Second, the certification of victims considered in this study was conducted by local governments. However, it is possible that specific groups of individuals may not have sought official recognition as disaster victims despite being impacted by the disaster. Therefore, the non-victims in this study may have included a few actual victims, which may have led to an underestimation regarding differences between victims and non-victims in our study. In addition, the quantitative measurement of mental and physical stress was not included in our data, although this variable could potentially influence the results of this study and should be included in future analyses. Third, we could not adjust for several important confounding factors, including environmental factors, physical and psychological stress levels, lifestyle factors such as smoking, clinical disease activity, and infectious and immunological conditions that might affect rheumatic diseases. Furthermore, data on health care-seeking behaviors, such as the frequency of medical consultations, were unavailable. It is possible that an increased frequency of health care visits among victims provided more opportunities for diagnosis and prescription, and this remains a potential unmeasured confounder. Fourth, information on deaths and transfers was excluded from the database. The analysis included all prescription data without excluding cases of death or transfer out of the area. This limitation may have led to an underestimation of the prescription rates because individuals who died or moved away during the study period were still accounted for in the denominator but could not be the source of new prescriptions. Fifth, regarding the statistical approach, alternative methods such as interrupted time series or difference-in-differences, potentially offering a more robust evaluation of the disaster’s impact, were considered. However, these were not applied because of the low monthly new MTX prescription event counts, especially pre-disaster, which made it challenging to meet model assumptions and ensure stable analyses. Finally, the potential for an increase in MTX prescriptions among victims, caused by the exemption of out-of-pocket medication costs, warrants consideration. However, given the relatively low drug prices and co-payment rates in Japan ^(17)^, along with the low price elasticity of outpatient care in the Japanese health insurance system (ranging from −0.125 to −0.076) ^(18)^, the increase in health care demand because of the exemption from medical expenses is limited and not considered to contribute to the rise in MTX prescriptions.

In conclusion, the victims of the 2018 Japan Flood were more likely to be prescribed MTX for the first time. This significant association may highlight the need for clinicians and policymakers to anticipate and prepare for an increased demand for specialized rheumatology care after major disasters. Further research is needed to draw a more robust conclusion.

## Article Information

### Acknowledgments

We are grateful to Editage (www.editage.jp) for providing English language editing.

### Author Contributions

Genki Kidoguchi contributed to the conception, design, analysis, interpretation of data, and drafting of the manuscript. Shuhei Yoshida contributed to the analysis, acquisition of data, interpretation of data, and critical revision of the manuscript. Tomohiro Sugimoto contributed to the design and critical revision of the manuscript. Shintaro Hirata contributed to design and critical revision of the manuscript. Masatoshi Matsumoto contributed to the design, acquisition of data, drafting of the manuscript, supervision, and critical revision of the manuscript. All authors read and approved the final manuscript.

### Conflicts of Interest

Shintaro Hirata received speaker fees, consultancy fees, research grants, and honoraria from AbbVie, Asahi-Kasei Pharma, Astellas, AstraZeneca, Ashima, Bristol Myers Squibb, Boehringer Ingelheim, Chugai, Daiichi-Sankyo, Eisai, Gilead Sciences, Glaxo Smithkline, Eli Lilly, Janssen, Novartis, Nippon Shinyaku, Otsuka, Pfizer, Taisho, Tanabe-Mitsubishi, and UCB. All other authors declare no conflicts of interest.

### Informed Consent

The requirement for informed consent was waived because the NDB data have been anonymized.

### Availability of Data and Material

Data cannot be shared because of restrictions mandated by the Ministry of Health, Labor and Welfare.

### IRB Approval Code and Name of the Institution

Ethical approval was granted by the Ethics Committee for Epidemiological Research at Hiroshima University (Ref. no. E-1389). The study was performed in accordance with the principles laid down in the Declaration of Helsinki.

### Previous Postings and Presentations

A preprint version of this manuscript was previously posted on Authorea (DOI: 10.22541/au.173711171.12516474/v1).

## Supplement

Supplementary Material Supplementary Figure 1. Kaplan-Meier failure curves for the participants newly prescribed with bDMARDs.
The Kaplan-Meier curve depicts the incidence of new bDMARDs prescription among MTX-treated participants within a span of 12 months after the disaster among victims and non-victims.
bDMARD: biological disease-modifying antirheumatic drugs; MTX: methotrexate.
Supplementary Figure 2. Kaplan-Meier failure curves for the participants newly prescribed with csDMARDs.
The Kaplan-Meier curve depicts the incidence of new csDMARDs prescription among MTX-treated participants within a span of 12 months following the disaster among victims and non-victims.
csDMARD: conventional synthetic disease-modifying antirheumatic drugs; MTX: methotrexate.
Supplementary Figure 3. Kaplan-Meier failure curves for the participants newly prescribed or injected with glucocorticoids.
The Kaplan-Meier curve depicts the incidence of new glucocorticoid prescription or injection among MTX-treated participants within a span of 12 months after the disaster among victims and non-victims.
MTX: methotrexate.

## References

[1] Germain V, Scherlinger M, Barnetche T, et al. Role of stress in the development of rheumatoid arthritis: a case-control study. Rheumatology (Oxford). 2021;60(2):629-37. 32533144 10.1093/rheumatology/keaa216

[2] Gravallese EM, Firestein GS. Rheumatoid arthritis - common origins, divergent mechanisms. N Engl J Med. 2023;388(6):529-42. 36780677 10.1056/NEJMra2103726

[3] Yoshida S, Kashima S, Matsumoto M. The effect of the 2018 Japan Floods on cognitive decline among long-term care insurance users in Japan: a retrospective cohort study. Environ Health Prev Med. 2021;26(1):113. 34856925 10.1186/s12199-021-01038-9 PMC8903631

[4] Okazaki Y, Yoshida S, Kashima S, et al. Impact of the 2018 Japan Floods on benzodiazepine use: a longitudinal analysis based on the National Database of Health Insurance Claims. Soc Psychiatry Psychiatr Epidemiol. 2022;57(12):2411-21. 35474395 10.1007/s00127-022-02289-9

[5] Okazaki Y, Yoshida S, Kashima S, et al. Increased prescriptions for irritable bowel syndrome after the 2018 Japan Floods: a longitudinal analysis based on the Japanese National Database of Health Insurance Claims and Specific Health Checkups. BMC Gastroenterol. 2022;22(1):263. 35619078 10.1186/s12876-022-02342-6 PMC9137058

[6] Okazaki Y, Yoshida S, Kashima S, et al. Impact of the 2018 Japan Floods on prescriptions for migraine: a longitudinal analysis using the National Database of Health Insurance Claims. Headache. 2022;62(6):657-67. 35467012 10.1111/head.14301

[7] Ochi S, Kato S, Leppold C, et al. Can a disaster affect rheumatoid arthritis status? A retrospective cohort study after the 2011 triple disaster in Fukushima, Japan. Int J Rheum Dis. 2018;21(6):1254-62. 29700971 10.1111/1756-185X.13301

[8] Tomio J, Sato H, Mizumura H. Impact of natural disasters on the functional and health status of patients with rheumatoid arthritis. Mod Rheumatol. 2011;21(4):381-90. 21267755 10.1007/s10165-011-0414-y

[9] Regarding exemption of the copayments for people affected by the 2018 Japan floods (No. 25) (Government Announcement) [Internet]. Ministry of Health, Labour and Welfare, Japan. 2010 [cited 2021 Aug 6]. Available from: https://www.mhlw.go.jp/content/10600000/000376815.pdf

[10] Kojima M, Nakayama T, Tsutani K, et al. Epidemiological characteristics of rheumatoid arthritis in Japan: prevalence estimates using a nationwide population-based questionnaire survey. Mod Rheumatol. 2020;30(6):941-7. 31625435 10.1080/14397595.2019.1682776

[11] Kubota K, Kamijima Y, Sato T, et al. Epidemiology of psoriasis and palmoplantar pustulosis: a nationwide study using the Japanese national claims database. BMJ Open. 2015;5(1):e006450. 10.1136/bmjopen-2014-006450 PMC4298108 25588781

[12] Asai S, Suzuki M, Hara R, et al. Comparison of effectiveness of methotrexate in patients with late-onset versus younger-onset rheumatoid arthritis: real-world data from an inception cohort in Japan (NICER-J). Mod Rheumatol. 2024;34(5):892-9. 38491996 10.1093/mr/roae027

[13] Kamiya K, Ohtsuki M. Epidemiological survey of patients with psoriatic arthritis in the Japanese Society for Psoriasis Research from 2017 to 2020. J Dermatol. 2023;50(1):12-25. 36261862 10.1111/1346-8138.16603 PMC10092149

[14] Gelfand AA, Halker Singh RB, Robbins MS. Worsening migraine: another casualty of natural disasters. Headache. 2022;62(6):645-7. 35670096 10.1111/head.14325

[15] de Brouwer SJM de, van Middendorp H van, Stormink C, et al. Immune responses to stress in rheumatoid arthritis and psoriasis. Rheumatology (Oxford). 2014;53(10):1844-8. 24850878 10.1093/rheumatology/keu221

[16] Malmström V, Catrina AI, Klareskog L. The immunopathogenesis of seropositive rheumatoid arthritis: from triggering to targeting. Nat Rev Immunol. 2017;17(1):60-75. 27916980 10.1038/nri.2016.124

[17] Mulcahy AW, Edenfield N, Becerra-Ornelas AU. International prescription drug price comparisons: current Empirical Estimates and Comparisons with Previous Studies. Rand Health Q. 2024;11(1):5. 10.7249/rhq-11-03-05 PMC11147645 38855386

[18] Sawano K. Co-payment, coinsurance rate and the elderly care in Japan. Iryo To Shakai. 2000;10(2):115-38.

