# Supplementary material for: Impact of the 2018 Japan Floods on Methotrexate and Antirheumatic Drug Prescriptions: A Longitudinal Analysis of the Japanese National Database
Source: JMA J. 2025 Nov 21;9(1):106–14. doi: 10.31662/jmaj.2025-0258 (PMC12889483; doi:10.31662/jmaj.2025-0258)
Supplement: Supplementary Material — Supplementary Figure 1. Kaplan-Meier failure curves for the participants newly prescribed with bDMARDs. The Kaplan-Meier curve depicts the incidence of new bDMARDs prescription among MTX-treated participants within a span of 12 months after the disaster among victims and non-victims. bDMARD: biological disease-modifying antirheumatic drugs; MTX: methotrexate. Supplementary Figure 2. Kaplan-Meier failure curves for the participants newly prescribed with csDMARDs. The Kaplan-Meier curve depicts the incidence of new csDMARDs prescription among MTX-treated participants within a span of 12 months following the disaster among victims and non-victims. csDMARD: conventional synthetic disease-modifying antirheumatic drugs; MTX: methotrexate. Supplementary Figure 3. Kaplan-Meier failure curves for the participants newly prescribed or injected with glucocorticoids. The Kaplan-Meier curve depicts the incidence of new glucocorticoid prescription or injection among MTX-treated participants within a span of 12 months after the disaster among victims and non-victims. MTX: methotrexate. [file 2433-3298-9-1-0106-s001.pdf]

Supplementary Table 1. Baseline Characteristics of Eligible Participants.

		Victims	Non-victims	All	ASD
All participants, n		31,235	4,968,894	5,000,129	
Age classification, n (%)	20–44	6,684 (21.4%)	1,712,322 (34.5%)	1,719,006	0.295
	45–64	8,155 (26.1%)	1,460,595 (29.4%)	1,468,750	0.074
	65-	16,396 (52.5%)	1,795,977 (36.1%)	1,812,373	0.335
Sex, n (%)	Male	13,583 (43.5%)	2,304,674 (46.4%)	2,318,257	0.058
	Female	17,652 (56.5%)	2,664,220 (53.6%)	2,681,872	0.058
Total MTX prescription before the disaster, n (%)		229 (0.73%)	26,499 (0.53%)	26,728	0.025

Data were presented as n (%).

An ASD of < 0.1 was considered to indicate a negligible difference between the groups.

Abbreviations: ASD, absolute standardized difference; MTX, methotrexate.

Supplementary Table 2. Adjusted Hazard Ratio and 95% Confidence Intervals for a New MTX Prescription in MTX-naïve Group.

	aHR	95% CI	p-value
Victims (Ref = non-victims)			
MTX	1.83	1.37-2.46	p<0.001

Details of adjusted variables are described in the Method section.

Abbreviations: MTX, methotrexate; aHR, adjusted hazard ratio; CI, confidence interval.

Supplementary Table 3. Adjusted Hazard Ratio and 95% Confidence Intervals for a New Prescription of Other Antirheumatic Drugs.

	aHR	95% CI	p-value
Victims (Ref = non-victims)			
bDMARDs	1.98	0.88–4.45	p=0.100
csDMARDs	0.94	0.42–2.10	p=0.885
Glucocorticoid	1.39	0.81–2.41	p=0.235
Any of the other antirheumatic drugs	1.21	0.80-1.82	p=0.369

"Any of other antirheumatic drugs" refers to participants who were prescribed bDMARDs, csDMARDs, or glucocorticoids. Details of adjusted variables are described in the Method section.

Abbreviations: aHR, adjusted hazard ratio; DMARDs, Disease modified antirheumatic drugs; bDMARDs, biological DMARDs; csDMARDs, conventional synthetic DMARDs; CI, confidence interval.

Supplementary Figure 1.

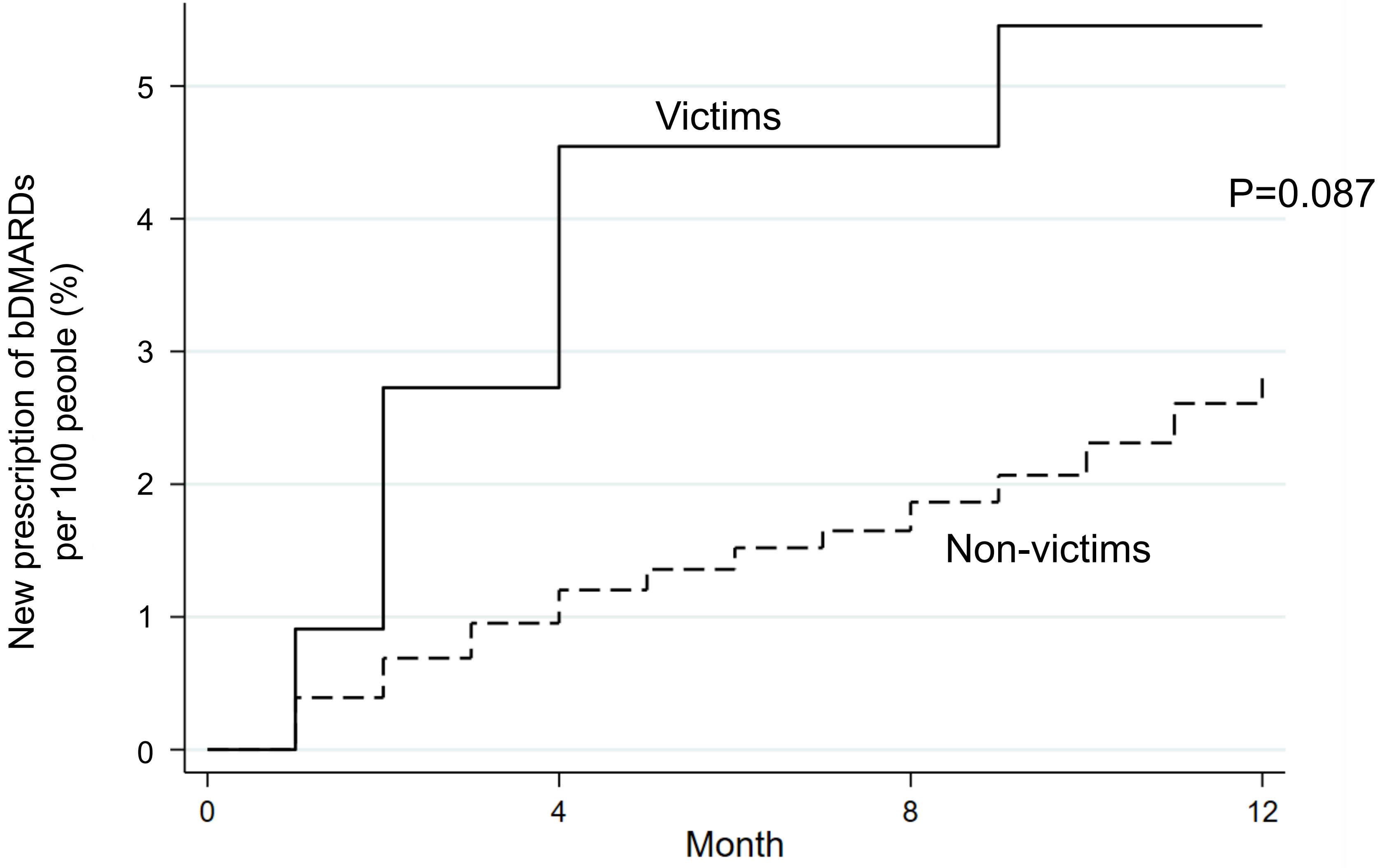

Number at risk				
Non-victims	14798	14657	14554	14412
Victims	110	*	*	*

Supplementary Figure 2.

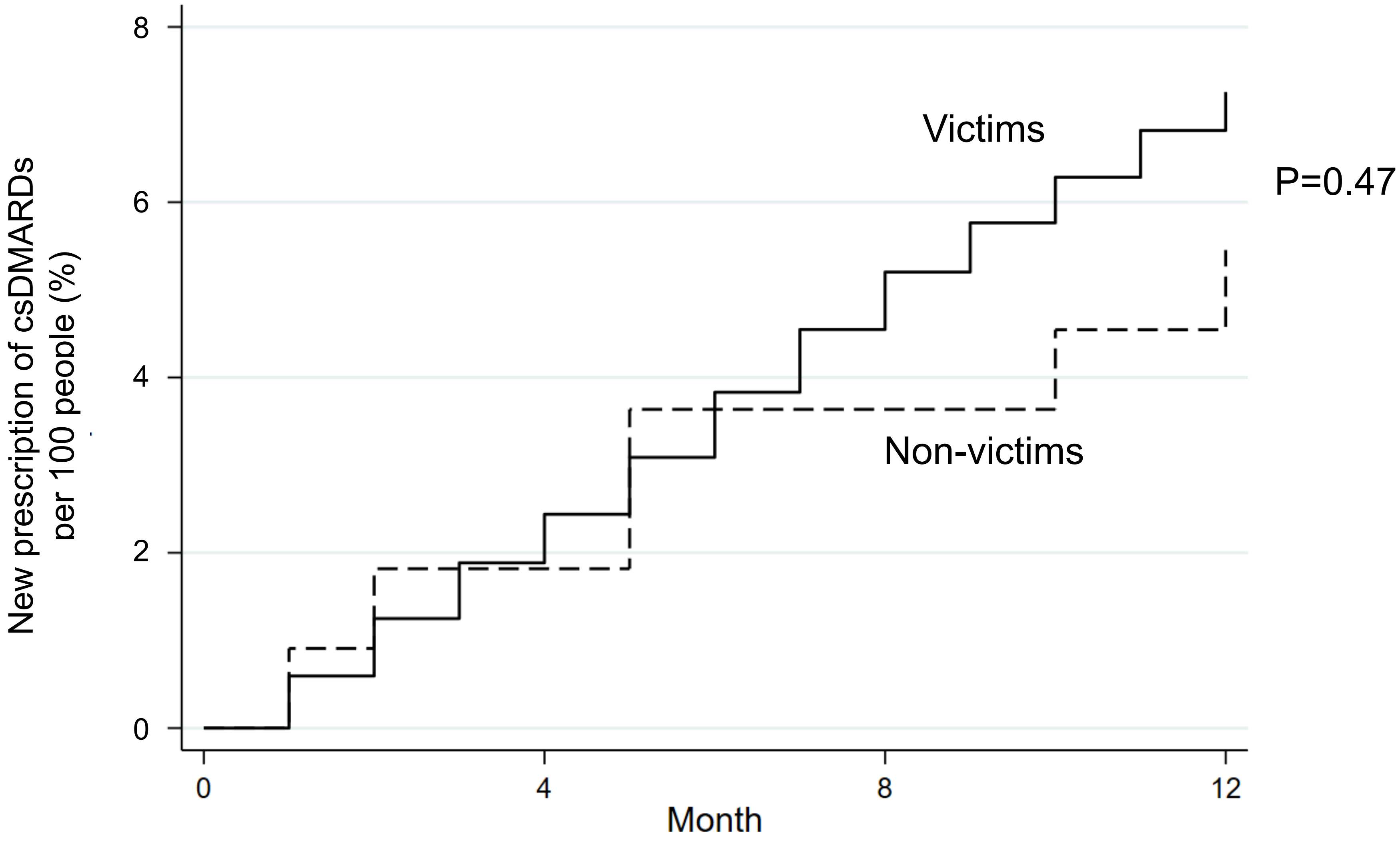

Number at risk				
Non-victims	14798	14519	14125	13789
Victims	110	*	*	*

Supplementary Figure 3.

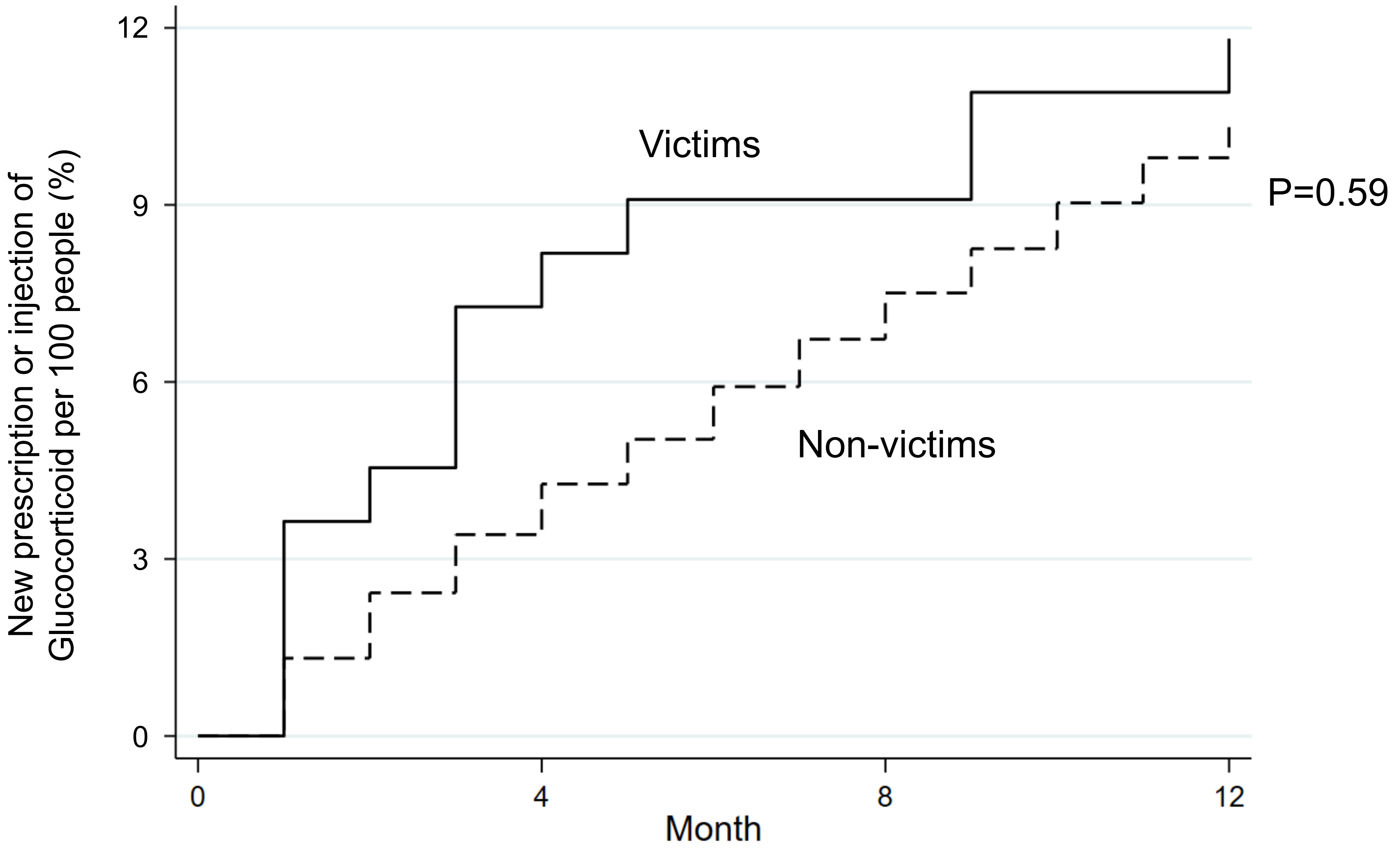

Number at risk				
Non-victims	14798	14293	13803	13348
Victims	110	*	*	*
